# Vesicular Stomatitis Virus Detected in Biting Midges and Black Flies during the 2023 Outbreak in Southern California

**DOI:** 10.3390/v16091428

**Published:** 2024-09-07

**Authors:** Stacey L. P. Scroggs, Dustin A. Swanson, Taylor D. Steele, Amy R. Hudson, Lindsey M. Reister-Hendricks, Jessica Gutierrez, Phillip Shults, Bethany L. McGregor, Caitlin E. Taylor, Travis M. Davis, Nadine Lamberski, Kristen A. Phair, Lauren L. Howard, Nathan E. McConnell, Nikos Gurfield, Barbara S. Drolet, Angela M. Pelzel-McCluskey, Lee W. Cohnstaedt

**Affiliations:** 1 Arthropod-Borne Animal Diseases Research Unit, Agricultural Research Service, United States Department of Agriculture, Manhattan, KS 66502, USA; taylor.steele@usda.gov (T.D.S.); amy.hudson@usda.gov (A.R.H.); lindsey.reister-hendricks@usda.gov (L.M.R.-H.); jessica.gutierrez@usda.gov (J.G.); phillip.shults@usda.gov (P.S.); bethany.mcgregor@usda.gov (B.L.M.); caitlin.taylor@usda.gov (C.E.T.); travis.davis2@usda.gov (T.M.D.); barbara.drolet@usda.gov (B.S.D.); 2 Center for Grain and Animal Health Research, Agricultural Research Service, United States Department of Agriculture, Manhattan, KS 66502, USA; dustin.swanson@usda.gov; 3 San Diego Zoo Wildlife Alliance, Safari Park, Escondido, CA 92027, USA; nllamberski@sdzwa.org (N.L.); kphair@sdzwa.org (K.A.P.); lhoward@peeltx.com (L.L.H.); 4 Peel Therapeutics, Salt Lake City, UT 84101, USA; 5 San Diego County Vector Control, San Diego, CA 92123, USA; nathan.mcconnell@sdcounty.ca.gov (N.E.M.); nikos.gurfield@sdcounty.ca.gov (N.G.); 6 Animal and Plant Health Inspection Service, Veterinary Services, United States Department of Agriculture, Fort Collins, CO 80526, USA; angela.m.pelzel-mccluskey@usda.gov; 7 Foreign Arthropod-Borne Animal Diseases Research Unit, National Bio- and Agro-Defense Facility, Agricultural Research Service, United States Department of Agriculture, Manhattan, KS 66502, USA; lee.cohnstaedt@usda.gov

**Keywords:** vesicular stomatitis New Jersey virus, *Culicoides*, *Simulium*, California, VSV, VSNJV, vector surveillance, arthropod-borne virus

## Abstract

Vesicular stomatitis (VS) is a viral disease that affects horses, cattle, and swine that is transmitted by direct contact and hematophagous insects. In 2023, a multi-state outbreak of vesicular stomatitis New Jersey virus (VSNJV) occurred in California, Nevada, and Texas, infecting horses, cattle, and rhinoceros. To identify possible insect vectors, we conducted insect surveillance at various locations in San Diego County, CA, including at a wildlife park. CO_2_ baited traps set from mid-May to mid-August 2023 collected 2357 *Culicoides* biting midges and 1215 *Simulium* black flies, which are insect genera implicated in VSNJV transmission. Insects were pooled by species, location, and date, then tested for viral RNA. Nine RNA-positive pools of *Culicoides spp.* and sixteen RNA-positive pools of *Simulium spp* were detected. Infectious virus was detected by cytopathic effect in 96% of the RNA-positive pools. This is the first report of VSNJV in wild-caught *C. bergi*, *C. freeborni*, *C. occidentalis*, *S. argus*, *S. hippovorum*, and *S. tescorum.* The vector competency of these species for VSNJV has yet to be determined but warrants examination. Active vector surveillance and testing during disease outbreaks increases our understanding of the ecology and epidemiology of VS and informs vector control efforts.

## 1. Introduction 

Vesicular stomatitis virus (VSV, *Rhabdoviridae*, *Vesiculovirus*) is the causative agent of vesicular stomatitis (VS) [1,2]. In the Americas, two serotypes of VSV, vesicular stomatitis New Jersey virus (VSNJV) and vesicular stomatitis Indiana virus (VSIV), predominantly cause disease in horses and cattle [1,2]. Clinical signs include blister-like lesions on the mouth, tongue, nostrils, coronary bands, and teats and are difficult to distinguish from those that present in foot and mouth disease (FMD) [1,2,3]. Because of its clinical similarity to FMD, VSV is also a reportable disease that requires a mandatory quarantine lasting 14 days after the last lesioned animal presents with clinical signs on a property. Quarantine leads to additional financial impacts due to limited animal movement [4,5]. On dairy farms, VS decreases milk production which results in major economic losses [6,7,8].

VSV is primarily transmitted by hematophagous insects but can also be transmitted by direct contact with infected animals and fomites [9]. Several arthropods have been implicated in VSV transmission [10], but vector competence has only been experimentally demonstrated for a handful of species of *Culicoides* biting midges [11,12,13], *Simulium* black flies [14,15,16,17,18,19], *Lutzomyia* sand flies [20,21,22,23], and *Aedes* mosquitoes [24,25,26,27,28]. VSV can also be transmitted between insects via co-feeding [29] and venereal transmission [30]. Field collections of insects during an active outbreak provide insight into the complex ecological dynamics of virus transmission and highlight possible vector species [4,18,31,32,33,34]. Due to the limited number of colonies available for these vector groups, field collections provide critical information for incriminating novel vectors and can be used to fulfill two of the four World Health Organization vector incrimination criteria; specifically, the recovery of virus from wild collected specimens and showing an association between infected arthropods and the affected vertebrate populations [35].

VS is endemic in Mexico and Central America; but about every 4–10 years, an endemic strain from Mexico moves northward into the United States [36]. These incursion events are most often caused by VSNJV [1,2]. Before 2023, VS outbreaks occurred in southwestern or Rocky Mountain states and did not extend to California with the exception of 1982 when infected cattle from Idaho were sold to several dairies in the San Joaquin Valley [8,37]. In May of 2023, the National Veterinary Services Laboratories (NVSL), Ames, IA, USA, confirmed VSNJV in an equine sample taken from a premises in San Diego County, California [38]. Between May 2023 and January 2024, a total of 319 VSV-affected premises were identified across 19 counties in California (*n* = 316), Nevada (*n* = 1), and Texas (*n* = 2) [39]. The primary host species impacted by the outbreak was equine (97.5% of VSV-affected premises), with cattle (2.8%) and rhinoceros (0.3%) also infected [39,40]. 

The emergence of VS in California is a major agricultural concern. California has 5.2 million cows and 477,400 horses, representing a total economic value of USD 14 billion and USD 9.5 billion, respectively [41,42,43]. Outbreaks of VS in California could drastically damage the dairy, beef, and equine industries. Animal wellbeing for non-livestock or non-equine hosts, such as the southern white rhinoceros and the greater one-horned rhinoceros, is an additional concern [40]. A total of 63 species of *Culicoides* biting midges and 76 species of *Simulium* black flies inhabit California [44,45,46]. Many of these species are likely not vectors of VSV as some do not blood feed, do not feed on competent hosts, or their ecology does not coincide with the epidemiology of VS. Understanding which species are competent vectors is a major gap in the transmission model of VS. We sought to identify possible insect vectors involved in VSNJV transmission during the 2023 outbreak by collecting biting midges and black flies from multiple locations across San Diego County and testing them for the presence of VSNJV. We also examined climatic and environmental conditions in 2023 to assess what conditions may have favored the first non-human-mediated outbreak of VS in California. 

## 2. Materials and Methods

### 2.1. Insect Collections

Collection of biting flies was performed by the San Diego County Vector Control Program (SDVCP). As part of regular mosquito surveillance and monitoring efforts for aerial larvicide operations, encephalitis virus surveillance (EVS) traps (BioQuip, Rancho Dominguez, CA, USA) were set biweekly near riparian areas and/or salt marsh lagoons with a history of mosquito production. Additional traps, including Reiter/Cummings Style Gravid Traps (BioQuip, BG (Biogents AG, Regensburg, Germany; with or without CO_2_), and EVS (with or without BG trap skin lure)), were also set in response to citizen complaints on an as-needed basis. Traps were set in the afternoon, picked up the following morning, and contents were frozen at −70 °C. Starting with insects collected on 23 May 2023, mosquitoes were removed, and other biting flies were stored at −70 °C and then shipped dry on dry ice to the USDA Center for Grain and Animal Health Research in Manhattan, KS, USA.

**Figure 1:**
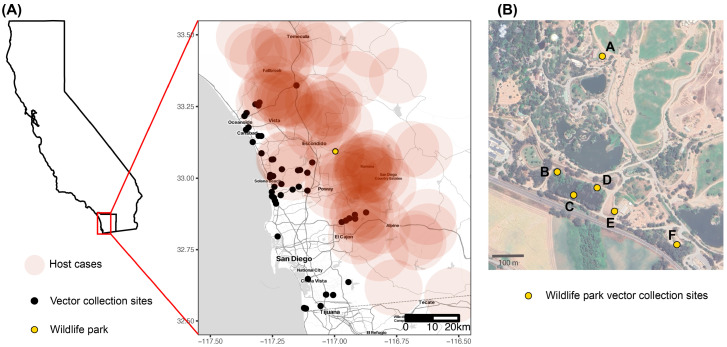
Insect collection sites in San Diego County, California, USA. (**A**) Location of insect collection sites (black circles) and the wildlife park (yellow circle). The red highlighted box indicates the area of San Diego County (entire county boundary in black) where sampling was conducted. The red circles indicate a 10 km buffer around confirmed or suspected VS premises. (**B**) Trap locations (*n* = 6; A–F) at the wildlife park.

Six EVS traps were deployed weekly at the Wildlife Safari Park by SDVCP starting on 16 June 2023. Two traps were located near the rhinoceros’ habitats (Figure 1B, traps A and E) and four were located near a drainage channel (Figure 1B, trap F) and overflow pond (Figure 1B, traps B, C, and D). After 8 weeks, the used EVS traps were no longer usable and were replaced with Stealth Traps (John W. Hock Company, Gainesville, FL, USA) as the EVS traps were no longer available for purchase. Trap locations remained the same. As above, all traps were set in the afternoon, picked up the following morning, and returned to the lab, where all contents were frozen dry at −70 °C and shipped to USDA on dry ice.

### 2.2. Vesicular Stomatitis Host Premises Data for San Diego County

Premises data with VS-positive animals were obtained from the USDA Animal Plant Health Inspection Service (APHIS) for confirmed positive and suspected cases in San Diego County in 2023 (*n* = 69). USDA-APHIS and State Animal Health Officials have mandated that VS is reportable by veterinarians to both state and federal authorities, resulting in reliable identification of case classification, host species, onset date, serotype, and premises latitude and longitude. Cases were classified as confirmed positive (*n* = 26) based on diagnostic confirmation of recent infection by multiple methods including PCR, virus isolation, and/or complement fixation testing (CFT) at NVSL. Additionally, some livestock premises were classified as suspect without full diagnostic confirmation based on presentation of vesicular lesions alone and were assumed to be infected (*n* = 42). There were 2 bovine premises, 1 exotic premises, and 65 horse premises classified as either confirmed positive or suspect in San Diego County. Onset dates represent the clinical onset of VS lesions or best estimate. To visually convey relative distances between host cases and vector collection sites, while still protecting stakeholder anonymity, a radial buffer of 10 km was applied to each premises location.

### 2.3. Morphological Identification of Insects

Specimens were sorted morphologically to the lowest taxonomic group possible and separated by sex on a chill table (−4 °C) to maintain the cold chain. Black flies were identified using the key of Adler et al. [45]. Biting midges were identified using the photographic wing atlas of Wirth et al. [47] and a subgeneric specific key [48]. Representative specimens for each species were saved to serve as vouchers. After sorting and identification, specimens were returned to −80 °C until viral screening could be performed.

### 2.4. Viral RNA Extraction and RT-qPCR for VSNJV Detection

Insects collected between 23 May and 15 August 2023 were processed and tested for the presence of VSNJV RNA. The testing cutoff date was two weeks after the last new VS-positive premises identified in San Diego County. The county was released from quarantine at the end of August 2023. In a Petri dish on wet ice, insects were pooled by species, trap ID, and collection date, with each pool containing seven or fewer individuals. Pools were homogenized in 500 μL of antibiotic media (M199E media with 2% fetal bovine serum (FBS), 400 μg/mL streptomycin, 400 U/mL penicillin, 200 μg/mL gentamycin, 25 μg/mL ciprofloxacin, and 5 μg/mL fungizone [49]) with three–four 2.4 mm stainless steel beads (Omni International, Kennesaw, GA, USA) using the Bead Ruptor Elite (Omni International, Kennesaw, GA, USA) for 4 min at 3.1 m/s; this was followed by centrifugation at 12,000× *g* for 8 min at 4 °C to pellet tissue debris. RNA was extracted from 200 μL cleared homogenate using the MagMAX™ CORE Nucleic Acid Purification Kit (Applied Biosystems; Thermo Fisher Scientific, Inc., Waltham, MA, USA) with the KingFisher X™ Apex System (Applied Biosystems; Thermo Scientific, Inc., Waltham, MA, USA) according to the manufacturer’s protocol. Extracted RNA was aliquoted and stored at −80 °C. Positive control VSNJV RNA was extracted from a viral stock of Vesiculovirus New Jersey (1982 bovine field isolate) [30]. 

VSNJV RNA was detected using the TaqMan Fast Virus 1-Step MasterMix (Applied Biosystems; Thermo Fisher Scientific, Inc., Waltham, MA, USA) on the 7500 Fast PCR Detection System (Applied Biosystems; Thermo Fisher Scientific, Inc., Waltham, MA, USA), targeting the L segment with the following primers: forward VSVNJ7274: 5′-TGATTCAATATAATTATTTTGGGAC-3, reverse VSVNJ7495 5′-AGG CTCAGAGGCATGTTCAT-3′, and probe: FAM-TTGCACACCAGAACATTCAA-3′-BHQ1 [30,50]. For amplification, the following conditions were used: reverse-transcription 1 cycle at 50 °C for 5 min, denaturing and polymerase activation at 95 °C for 20 s, and amplification: 40 cycles of 95 °C for 3 s and 60 °C for 30 s. All PCR reactions were conducted in duplicate and included a water negative control and a 10-fold dilution standard curve using extracted VSNJV RNA. Samples with a Ct value ≤ 36.6 were considered positive for VSNJV RNA [30,51]. 

Extracted RNA from VSNJV-positive pools was subject to whole-genome sequencing on the Illumina platform using random hexamer primers. Paired reads were aligned to a VSNJV reference genome (NJ061NME6) to identify VSNJV reads.

### 2.5. Molecular Identification of C. variipennis Complex

Due to morphological similarity among members of the *C. variipennis* complex, molecular identification was used to further differentiate species. DNA was extracted from the homogenized pools of VSNJV-positive samples morphologically identified as the *C. variipennis* complex using a Puregene Tissue Kit (Qiagen, Hilden, Germany). Amplification targeted a partial region of the COI using the LCO/HCO primers: 5′-3′–GGTCAACAAATCATAAAGATATTGG and 5′-3′–TAAACTTCAGGGTGACCAAAAAATCA [52]. PCR products were cleaned using an EXOSAP-IT kit (Applied Biosystems; Thermo Fisher Scientific, Inc., Waltham, MA, USA) and prepared for sequencing using a BigDye Terminator v.3.1 Cycle Sequencer Kit (Applied Biosystems; Thermo Fisher Scientific, Inc., Waltham, MA, USA). Sanger sequencing was performed using an Applied Biosystems 3500 Genetic Analyzer (Applied Biosystems; Thermo Fisher Scientific, Inc., Waltham, MA, USA). Chromatograms were cleaned and aligned using the software Geneious Prime software v.2023.0.4 (https://www.geneious.com). Additionally, paired reads from the whole-genome sequencing reactions in Section 2.4 were aligned to the cytochrome oxidase I (COI) gene for *Culicoides occidentalis* (GenBank Accession #OL604779.1). The consensus sequence of the reads mapping to the COI gene were extracted from each sample and input into the NCBI BLAST database (https://blast.ncbi.nlm.nih.gov/Blast.cgi; accessed May 2024) to identify the *Culicoides* species.

### 2.6. Infectious Virus Isolation

VSNJV RNA-positive pools were evaluated for infectious virus by inoculating 75% confluent monolayers of baby hamster kidney cells (BHK, ATCC, Manassas, VA, USA) in 12-well plates with 100 μL of the homogenized pools as described above. Plates were then incubated for 1 h at 37 °C, 5% CO_2_, with rocking every 15 min. After incubation, 1 mL of maintenance media (Eagle’s minimum essential medium with 2.2 g/L NaCHO_3_, 1× glutamax, and 10% FBS) was then added to each well. Plates were returned to the incubator and checked every 24 h for cytopathic effect (CPE). On 7 days post infection (dpi), cell monolayers were scraped and collected with supernatants then clarified via centrifugation. A volume of 100 μL clarified media was used to inoculate a fresh monolayer of BHK cells, 200 μL was extracted for RNA to confirm VSNJV, and the remaining was stored at −80 °C. Infectious virus was confirmed for the CPE+ samples by RT-qPCR, as above in Section 2.4. 

### 2.7. Environmental Data

Previous studies have identified anomalous temperature, precipitation, and vegetation greenness (Normalized Difference Vegetation Index, NDVI) leading up to equine VS lesion onset [53,54], presumably by influencing insect vectors’ development rates, larval habitat, microclimate refugia, and/or sugar resource availability. To better understand how the environment may have promoted the novel incursion and spread of VS in the San Diego region, we explored how these environmental conditions in 2023 differed from those in previous years. NDVI, derived from the Moderate-Resolution Imaging Spectroradiometer (MODIS) on the Terra satellite (MOD13Q1 version 6.1; [55]), was accessed through Google Earth engine [56] at a 250 m resolution and then area-averaged over the region of interest (−117.5 to −116.25° E and 32.5 to 33.5° N); the 16-day observations were then averaged by month. Higher NDVI values indicate greener conditions over the region. Monthly gridded precipitation and minimum and maximum temperatures from the Parameter–elevation Regressions on Independent Slopes Model (PRISM) climate group [57] were accessed through the prism package [58] in R studio [59] at a 4 km resolution; then, they were area-averaged over the region of interest from water year 2001 to 2023 (October 2000 to September 2023) to match the time period of NDVI. Minimum, maximum, and interquartile ranges for the monthly environmental values across water years 2001–2022 were plotted to visually compare with water year 2023 values.

### 2.8. Statistical Analyses

Two estimates of infection rate, the maximum likelihood estimate (MLE) and the minimum infection rate (MIR), were calculated for each species controlling for variable pool size using the R package PooledInfRate (CDC, version 1.6) [31,60,61]. Data were graphed using GraphPad Prism (version 10.1.1).

## 3. Results

### 3.1. Culicoides and Simulium Individuals Were Collected throughout San Diego County

**Figure 2:**
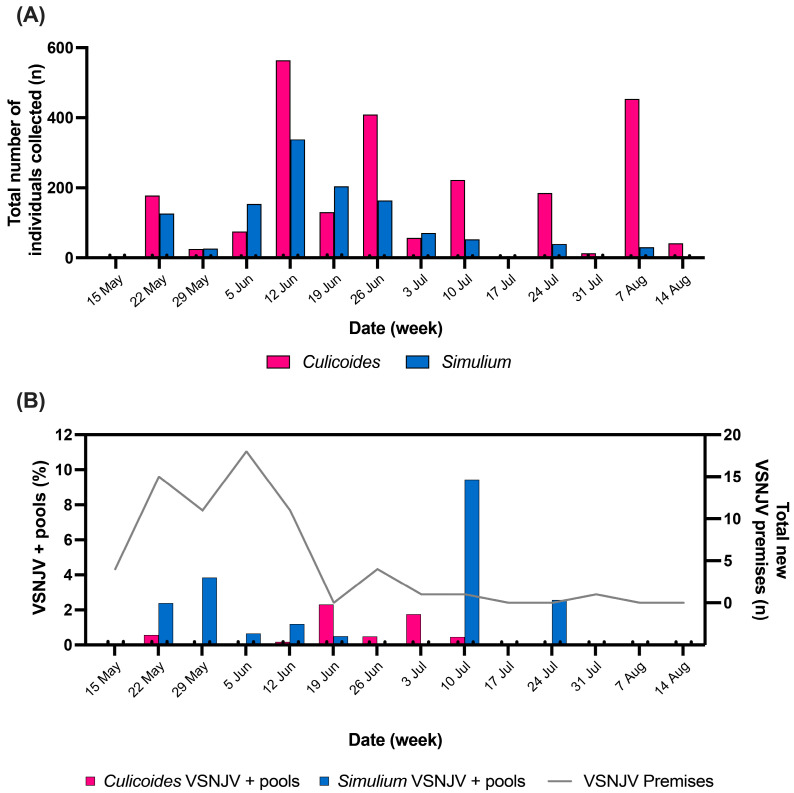
Insect collections, VSNJV-positive insect pools, and positive premises during the VSNJV 2023 outbreak in San Diego County. (**A**) Abundance of individual *Culicoides* (pink) and *Simulium* (blue) collected between May and August 2023. (**B**) Prevalence of *Culicoides* (pink) and *Simulium* (blue) VSNJV-positive pools and number of newly confirmed or suspected VSNJV-positive premises by date, as reported by APHIS (gray line).

*Culicoides* and *Simulium* were collected in San Diego County from 66 different locations (Figure 1A) between 22 May and 15 August 2023. A total of 2355 *Culicoides* and 1215 *Simulium* were collected. Four species of *Culicoides* and five species of *Simulium* were identified (Table 1). The most abundantly collected species were *C. freeborni*, *C. variipennis* complex, and *S. tescorum* (Table S1). *Simulium* were most numerous in late spring/early summer and peaked the week of 12 June. During the same week, a peak of *Culicoides* also occurred followed by a second peak the week of 7 August (Figure 2A). The abundance of both *C. freeborni* and *C. variipennis* complex peaked mid-June and again in early August, while *S. tescorum* peaked in mid-June only (Table S1). The peak date for confirmed or suspected VSV positive premises also occurred in early June (Figure 2B).

### 3.2. VSNJV RNA and Infectious Virus Detected in Culicoides and Simulium Pools

For viral screening, the trap collections were sorted by species, trapping location, and date into 529 *Culicoides* pools (average 4.5 individuals per pool) and 319 *Simulium* pools (average 3.8 individuals per pool) for VSV testing. All 850 pools were tested for VSNJV RNA via RT-qPCR and 25 VSNJV-positive pools (Ct ≤ 36.6) were identified (Table 1 and Table S2, Figure 2B). Positive pools were collected between the first week of trapping, 22 May, and the week of 24 July, with 68% collected before the start of July. This pattern of viral detection in vectors coincides with identification of the majority (96%) of VS-positive premises (Figure 2B, grey line). Most positive pools were *Simulium* (64%), specifically *S. argus* (*n* = 1), *S. hippovorum* (*n* = 3), *S. tescorum* (*n* = 9), and *S. vittatum* complex (*n* = 3). The *Culicoides* positive pools (36%) came from three species, *C. bergi* (*n* = 1), *C. freeborni* (*n* = 3), and *C. variipennis* complex (*n* = 5). The five VSNJV-positive pools that were morphologically identified as being from the *Culicoides variipennis* complex were sequenced and four were molecularly identified as *C. occidentalis*. Unfortunately, sequencing failed on the fifth pool and molecular identification was unsuccessful. The VSNJV positivity rate by species ranged from 1.4% for *C. freeborni* to 21.4% for the *S. vittatum* complex.

**Figure 3:**
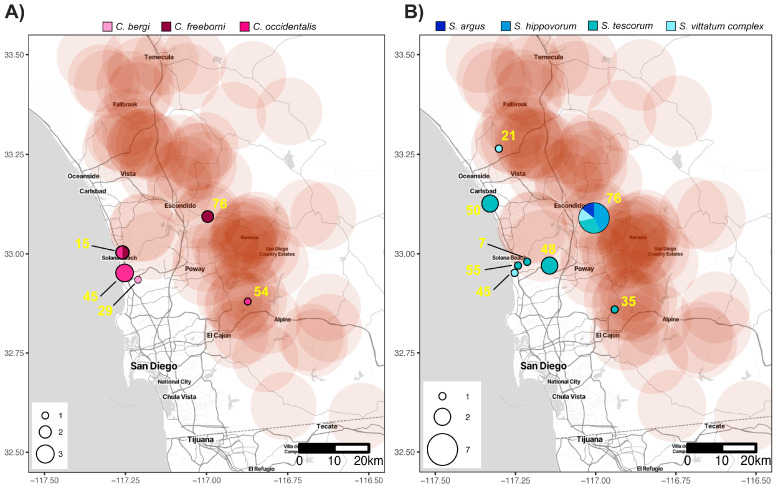
VS-positive premises and insect collections during the 2023 outbreak in San Diego County. Collection locations in San Diego County of VSNJV-positive *Culicoides* (**A**) and *Simulium* (**B**) pools. Circle size indicates the total number of VSNJV-positive pools collected at each location. Color of circles designates the insect species, as indicated in the legends. Trap sites are numbered in yellow. The large red circles indicate a 10 km buffer around confirmed or suspected VS-infected premises.

**Figure 4:**
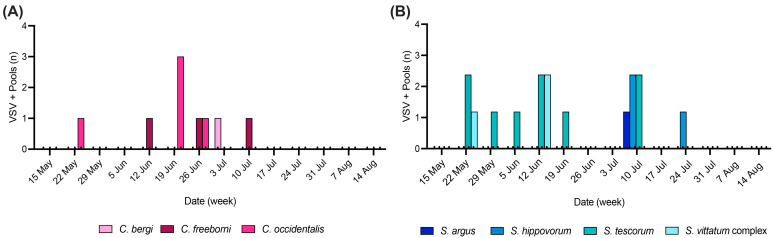
Collection dates of VSNJV-positive *Culicoides* (**A**) and *Simulium* (**B**) pools during the 2023 outbreak in San Diego County. Color designates the insect species, as indicated in the legends. VSNJV prevalence by species and date is located in Table S3.

*Culicoides* VSNJV-positive pools were collected from five locations—three coastal (trap sites 15, 29, and 45) and two inland (trap sites 54 and 76) (Figure 3A). The coastal pools (*n* = 6) accounted for 67% of the *Culicoides* VSVNJV-positive pools and contained *C. bergi* (*n* = 1), *C. freeborni* (*n* = 1), and *C. occidentalis* (*n* = 4). Most (67%) of the *Culicoides* VSNJV-positive pools were collected mid–late June (Figure 4A). The earliest VSNJV-positive *Culicoides* pool (*C. occidentalis*) was collected during the first week of trapping (22 May) and the last VSNJV-positive pool (*C. freeborni*) was collected the week of 10 July. Excluding *C. bergi*, which was not abundantly collected and had only one VSNJV-positive pool, the highest prevalence of VSNJV occurred in *C. occidentalis* pools (1.7%) (Table 1 and Table S3). The average distance to the closest VS-positive host premises was 6.3 km (+/−2.2 SE).

*Simulium* VSNJV-positive pools were collected from eight locations (Figure 3B), half of which were coastal (trap sites 7, 45, 50, and 55). The primary species with VSNJV-positive pools along the coast was *S. tescorum* (83%), while one pool (17%) was *S. vittatum* complex. The pools collected from inland trap sites 21, 35, 48, and 76 accounted for 69% of the *Simulium* VSNJV-positive pools. While positive pools of *S. tescorum* (*n* = 5) and *S. vittatum* (*n* = 2) were collected inland as well as on the coast, VSNJV-positive *S. argus* (*n* = 1) and *S. hippovorum* (*n* = 3) pools were only trapped inland. VSNJV-positive *Simulium* pools were caught consistently between May and July, especially *S. tescorum* (Figure 4B).

Positive pools of *S. vittatum* complex were collected in May and June, while positive pools of *S. argus* and *S. hippovorum* were collected only in July. While the VSNJV prevalence for pools of *S. bergi*, *S. hippovorum*, and *S. vittatum* ranged from 5.6% to 21.4%, the total number of pools tested for these species was fairly low (18, 17, 14) (Table 1 and Table S3). The prevalence of VSNJV in pools of *S. tescorum* was 3.9% from a total of 228 total pools tested. The average distance to the closest VS-positive host premises was 6.4 km (+/−1.6 SE).

Infectious virus was detected via cytopathic effect (CPE) after a single passage on baby hamster kidney (BHK) cells and confirmed with RT-qPCR (Table S1) for 24 out of the 25 VSNJV-positive pools. After an additional passage on BHK cells, only one VSNJV-positive pool was confirmed to be CPE-positive with RT-qPCR. All VSNJV-positive pools were negative for CPE in Vero cells. Whole-genome sequencing from all the positive pools was unsuccessful for VSNJV.

### 3.3. VSNJV-Positive Pools Were Collected at a Wildlife Park When Rhinoceros Were Symptomatic

**Figure 5:**
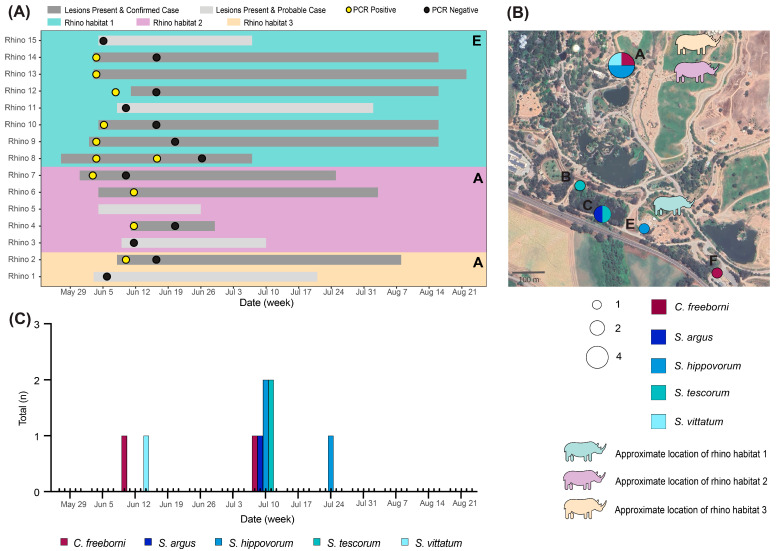
VSNJV-positive insect pools collected from 29 May to 21 August while rhinoceros were symptomatic. (**A**) Lesion onset and PCR-positive dates for probable and confirmed VS rhinoceros cases. Bars indicate the lesion time period for probable (light gray) and confirmed (dark gray) cases. Circles indicate when swabs from the rhinoceros were positive (yellow) or negative (black) for VSNJV RNA via RT-qPCR. Rhinoceros are categorized by habitat, indicated by the background color (teal, purple, and peach) and the closest trap to each habitat is specified by a letter in the top right corner (E, A). (**B**) Total number of VSNJV-positive pools by collection location (*n* = 6; A–F) and insect species at the wildlife park. Circle size indicates the total number of pools collected at each location that tested positive for VSNJV RNA. Color of circles indicates the insect species. Colored rhinoceros indicate the approximate location of the three rhinoceros habitats. (**C**) Species specific (*Culicoides* and *Simulium*) total number of VSNJV-positive pools by date collected at the wildlife park.

One trapping location was a wildlife park, where six traps were deployed (A–F; Figure 1B and Figure 5B) around the rhinoceros’ habitats. Fifteen rhinoceros with lesion date data were classified as a confirmed VS case based on a VSNJV-positive PCR or a probable case based on consistent clinical symptoms, but were not PCR confirmed (Figure 5A) [40]. Two species of rhinoceros reside at the wildlife park. *Ceratotherium simum simum*, or the southern white rhinoceros, are located in two enclosures near traps A and E (rhinos 3–15 in Figure 5A,B). *Rhinoceros unicornis*, or the greater one-horned rhinoceros, are in one enclosure near trap A (rhinos 1–2 in Figure 5A,B).

A total of 30 *Culicoides* (3 species sorted into 14 pools) and 131 *Simulium* (5 species sorted into 42 pools) were collected between the weeks of 12 June and 7 August 2023. Of the three species of *Culicoides* trapped at the wildlife park (*C. crepuscularis*, *C. freeborni*, *C. variipennis* complex), only two pools of *C. freeborni* from two trap sites tested positive for VSNJV RNA (Figure 5C). The VSNJV-positive *C. freeborni* pool collected the week of 12 June was trapped at trap A, while the second VSNJV-positive *C. freeborni* pool was collected the week of 10 July from trap F (Table 2). Of the 42 pools of *Simulium*, 7 tested positive for VSNJV (Figure 5B,C), including 4 species (*S. argus*, *S. hippovorum*, *S. tescorum*, *S. vittatum* complex). Four pools of *S. donovani* were tested, but VSNJV RNA was not detected in these samples. The one VSNJV-positive pool of *S. vittatum* complex was collected the week of 12 June from trap A (Table 2). Five VSNJV-positive pools were collected the week of 10 July from *S. argus* (*n* = 1; trap C), *S. hippovorum* (*n* = 2; traps A and E), and *S. tescorum* (*n* = 2; traps B and C). The last VSNJV-positive pool was collected the week of 24 July from *S. hippovorum* from trap A. Additionally, seven individuals of a single deer fly species (*Tabanidae)* collected with the midges and black flies all tested negative for VSNJV RNA.

The VS-confirmed rhinoceros tested PCR positive between 2 June and 17 June, a time frame that includes 12 June, which was the insect collection date with two VSNJV-positive pools. The majority of VSNJV-positive pools detected at the wildlife park were collected in July when many rhinoceros were still symptomatic, but 80% had cleared the virus, as confirmed by PCR.

### 3.4. Field Infection Rates

Two measures that estimate field infection rates, the minimum infection rate (MIR) and the maximum likelihood estimate (MLE), were calculated to estimate the total positive individual insects per 1000 in the population or the epidemiological impact for each potential vector species (Table 1) [31]. The *C. variipennis* complex and *S. tescorum* were the only two groups with significant MIR and MLE values (i.e., the 95% CI did not include 0). The MIR and MLE for the *C. variipennis* complex were both 4.4 infected individuals per 1000, while the MIR and MLE for *S. tescorum* were almost double at 8.5 and 8.6 infected individuals per 1000, respectively. The MIR and MLE for *C. freeborni*, while not significant, were the lowest estimates of all the species (2.9 for both) and had narrow confidence intervals (0–6.1 for both). The small number of positive pools in the other potential vector species resulted in MIR and MLE values with wide 95% confidence intervals that included 0.

### 3.5. Environmental Conditions in San Diego County before and during the Outbreak Were Atypical

**Figure 6:**
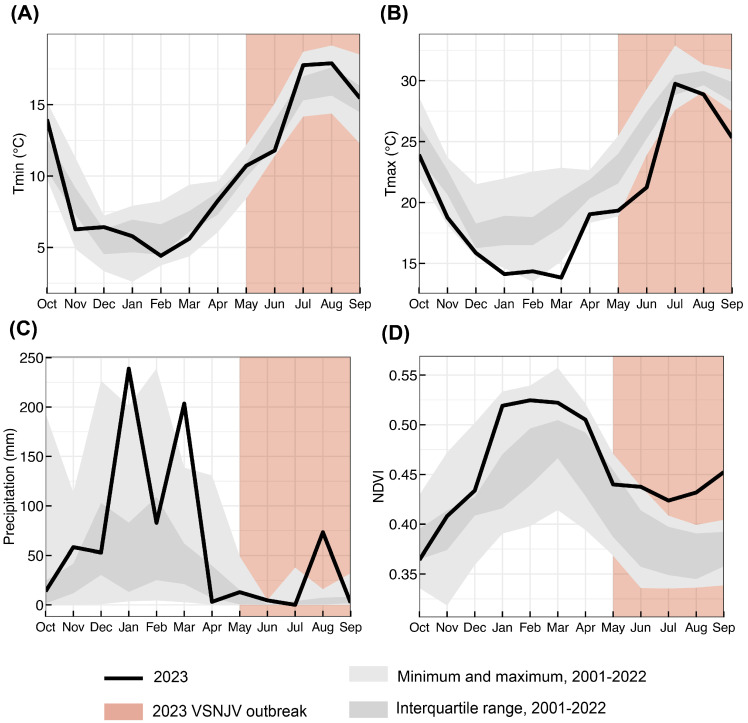
Environmental conditions leading up to and during the 2023 VS outbreak compared to previous 21-year ranges. Black lines indicate (**A**) minimum temperature, (**B**) maximum temperature, (**C**) precipitation, and (**D**) vegetation greenness (NDVI) for months in water year 2023 (spanning October 2022 to September 2023). Dark gray areas indicate the interquartile ranges for water years 2001–2022. Light gray areas indicate the minimum and maximum monthly values over the 2001–2022 period. Red rectangle from May to September indicates the 2023 VSNJV outbreak.

In 2023, the maximum temperatures were generally cooler leading up to and during the transmission season (Figure 6B), with minimum temperatures slightly warmer than typical in July and August during VS transmission (Figure 6A). Rainfall in January and March of 2023 was the highest over the 22 yr period, followed by large positive anomalies in August due to a tropical cyclone (Figure 6C). These precipitation anomalies drove increases in vegetation growth in January and caused anomalously higher greenness throughout the rest of the water year (i.e., 1 Oct. through 30 Sept) into the transmission season (Figure 6D). Cooler maximum temperatures during the summer transmission season may have also promoted increased vegetation. Cases occurred from May to August, and it is unlikely that the increased August precipitation influenced case numbers.

## 4. Discussion

In 2023, an unprecedented outbreak of VS occurred in California in the absence of animal movement, unlike the situation that led to the state’s last outbreak in 1982. It was first detected in San Diego County and ultimately spread widely throughout California, affecting horses, cattle, and two species of rhinoceros [39]. As has been utilized during previous outbreaks [4,18,31,32,33,34], opportunistic and targeted insect trapping provided crucial information about potential insect vectors involved with VSV transmission. Furthermore, pairing these data with environmental parameters can help to inform modeling efforts to improve predictive capabilities for future VSNJV outbreaks.

Of the seven biting-fly species that tested positive for VSNJV, six represent new records of VSV detection. For the black flies, only *S. vittatum* complex has been found positive in previous VS outbreaks in the US [4,18,33]. This study is the first documentation of VSV detection in *S. tescorum*, *S. argus*, and *S. hippovorum*. Many previous VSV detections in black flies have been from species within the subgenera *Psilozia* and *Psilopelmia* [14,15,16,17,29,31,32]. *Simulium argus* is a member of *Psilozia* and related to *S. vittatum*, a known competent vector of VSV [14,15,16,17]. Detection of VSV in *S. tescorum and S. hippovorum* are first reports for the subgenera *Aspathia* and *Hemicnetha*, respectively. For biting midges, previous positive detections during outbreaks have been found in *C. stellifer*, an unidentified species of the subgenus *Selfia*, and members of the *C. variipennis* complex, specifically *C. sonorensis* and *C. variipennis* [31,62,63]. Three members of the *C. variipennis* complex are known to be present in Southern California [57]. However, of these, only *C. sonorensis* has been experimentally shown to be competent for VSV transmission [11,12,13]. Additionally, VSV was isolated from field-collected *C. sonorensis* during both the 1982–83 outbreak in Colorado [62,63] and the 2019–2020 outbreak in Kansas [31]. Despite this species being quite prevalent in California and heavily associated with the affected hosts [64], almost all positive samples (4/5 pools) collected from the *C. variipennis* complex were molecularly identified as *C. occidentalis*. This is the first report of any arbovirus detected in *C. occidentalis* and the general consensus has been that this species is a poor vector [65]. However, this information is based on only two field populations with a single virus, and warrants further investigation given our findings. Further complicating this situation, the most accurate way to distinguish *C. occidentalis* from *C. sonorensis* is by using molecular markers [66]. The females of these species are morphologically very similar which is why they were designated as *C. variipennis* complex during the initial sorting. *Culicoides mullensi*, another species in the complex, was also detected in at least one VSNJV-negative sample. Therefore, we are unsure as to the exact composition of each VSNJV-negative pool that was morphologically identified as *C. variipennis* complex. Because of this uncertainty, the estimates of MIR and MLE for *C. occidentalis* could potentially be higher than what is reported here. Future studies that molecularly identify the *C. variipennis* complex species for each individual prior to pooling as well as experimental laboratory infections are needed to clarify the role that each cryptic species plays in VSNJV transmission. The detection of VSV in *C. bergi* and *C. freeborni* are the first detections in the subgenera *Diphaomyia* and *Culicoides,* respectively. For all newly implicated vector species (*C. bergi*, *C. freeborni*, *C. occidentalis*, *S. argus*, *S. hippovorum*, and *S. tescorum*), laboratory infections are needed to determine their competence for VSV.

Infection rates varied across the different species tested. Black flies had three of the highest infection rates tested (Table 1) with *S. vittatum* complex at 21.4%, *S. hippovorum* at 17.6%, and *S. argus* at 5.6%. For the *Culicoides*, only *C. bergi* at 5.6% was comparable to infection rates in black flies. However, the species with the highest infection rates were also some of the least abundant in trap collections. This is reflected in the wide and nonsignificant confidence intervals for the field population infection rates, which take number of pools and pool sizes into account. The most abundant species was the C. *variipennis* complex, accounting for 33% of all insects, collected followed by *S. tescorum* at 30%. However, *S. tescorum* had more than double (3.9%) the infection rate of *C. variipennis* complex (1.7%). Additionally, the significant population infection rates were also double for *S. tescorum* compared to *C. variipennis* complex (8.5 infected individuals per 1000 compared to 4.4 infected individuals per 1000). *Culicoides freeborni* was the third most abundant species collected in the traps and had an infection rate of 1.4%. The field population infection rate for *C. freeborni* was also not significant, but the confidence interval was smaller, highlighting the importance of adequate sample size in estimating infection rates in a population.

Virus detection depended primarily on RT-qPCR. Isolation of infectious virus and viral sequencing were attempted but less or not successful, respectively. Culturing live viruses from field-caught insects is notoriously difficult. To increase the likelihood of infectious virus detection, we maintained the cold chain during sample processing and shipment. However, multiple factors including insect size and inherent field infection variation could have contributed to the difficulty in obtaining isolates capable of sustained replication during cell culture passaging and whole-genome sequences.

Our results suggest that *S. tescorum* contributed the most to disease transmission during the 2023 outbreak in San Diego County. However, caution should be taken in trying to infer which families or species were potentially important in this VSV incursion event. Traps deployed to collect mosquitoes are not always effective in collecting black flies and midges [67,68]; thus, the numbers recorded here are likely not a complete representation of populations and their abundance in San Diego County. Additionally, the locations of mosquito traps across San Diego County were primarily located in urban areas rather than the rural or agricultural regions where VS-positive host premises were largely located (Figure 1A). Infection rates also could be a reflection of host preferences of the collected flies. Many of the species collected are known to feed on large mammals like horses, cattle, and sheep [45,69,70]. These insect species may have ingested virus from feeding on infectious hosts, and even become infected, but may not be competent vectors for VSV. Bloodmeal analyses from blood-fed insects can be used to identify host associations [69,71]. There was no observable blood present in the samples from the current study, so bloodmeal analyses were not conducted. Mosquitoes collected in the traps were maintained for other pathogen testing and were not tested for VSNJV in this study. Therefore, it is unclear whether mosquitoes may have played a role in the transmission cycle of VSV in California.

Environmental anomalies in 2023 were consistent with expectations of a VS outbreak from the literature and our understanding of VS vector capacity. Peters et al. [53] have found similar environmental anomalies leading up to case onset in previous outbreak years (2004, 2005, 2014, and 2015) in the more central–western states of Colorado, New Mexico, Texas, and Wyoming. Incursion has coincided with increased vegetation greenness at onset and up to 8 months prior in these other systems [53]; similarly, it was high at onset and up to 6 months prior, as well as through the transmission period in this study. Other significant environmental predictor variables include increased rainfall four months prior to onset, and decreased minimum temperatures one month prior to onset [53]. While we did not find lower minimum temperatures, the maximum temperatures were anomalously low in April 2023, one month prior to onset (Figure 6B).

Positive precipitation anomalies paired with cooler maximum temperatures likely resulted in areas of wet soil and flowing water, which may have increased the larval habitats for vector species. Biting midge larval habitats consist primarily of wet soils or other substrates [72,73,74], while black fly larvae require flowing water to filter feed [45]. Surface runoff and streamflow were not explicitly included in this study as they are most relevant at finer spatial scales; however, significant correlations with VSV onset, lower surface runoff, and higher streamflow at equine premises has been shown [53]. Vesicular stomatitis cases generally begin at sites near flowing water [75,76,77] and after a return to base flow in neighboring streams [54], which matches with these 2023 cases beginning after peaks in spring precipitation and likely streamflow. Cooler maximum temperatures could also help keep river temperatures cooler for more ideal black fly larval habitat [45]. Higher vegetation greenness may have also influenced cooler temperatures along water ways, increased food availability in flowing streams, and increased microclimate availability as leaves and plant tissue provide shelter for vectors. High NDVI may also be a proxy for areas of wetter soil (*Culicoides*) and increased streamflow (*Simulium*).

The 2023 incursion of VS into southern California initiated without the movement of infected livestock was an unprecedented event for the state. Incursions of VSV from Mexico into the southwestern and plains states of the United States occurs roughly every 4–10 years [36]. It remains to be seen if the California incursion was an outlier, possibly driven by abnormal weather conditions that bolstered larval habitats, or if the geography of VSV incursions is expanding. Additionally, phylogenetic data are needed from the 2023 outbreak to elucidate the origin of the incursion virus. The identification of six new species as possible vectors of VSV highlights the need for further studies on vector competence for VSV to better understand the epidemiology of VS and provide greater predictive power as to when and how VS incursions might occur.

## Figures and Tables

**Table 1:** VSNJV detections in *Culicoides* and *Simulium* pools via RT-qPCR and field infection rates.

Species	Total N (Pools)	VSNJV + Pools (%)	Ct Range	MIR (95% CI)	MLE (95% CI)
*Culicoides bergi*	75 (18)	1 (5.6)	35.61	8.0 (0–23.6)	8.0 (0–23.8)
*Culicoides crepuscularis*	1 (1)	0	-	NA	NA
*Culicoides freeborni*	1048 (222)	3 (1.4)	34.8–35.6	2.9 (0–6.1)	2.9 (0–6.1)
*Culicoides variipennis* complex/ Culicoides occidentalis *	1148 (242)	5 (1.7)	29.1–36.6	4.4 (0.5–8.2)	4.4 (0.6–8.3)
*Simulium argus*	38 (18)	1 (5.6)	35.5	26.3 (0–77.2)	26.7 (0–78.6)
*Simulium donovani*	42 (16)	0	-	NA	NA
*Simulium hippovorum*	36 (17)	3 (17.6)	28.2–36.2	83.3 (0–173.6)	90.0 (0–188.9)
*Simulium tescorum*	1055 (228)	9 (3.9)	29.2–36.6	8.5 (3.0–14.1)	8.6 (3.0–14.3)
*Simulium vittatum* complex	37 (14)	3 (21.4)	35.3–35.9	81.1 (0–169.0)	88.9 (0–186.8)

* VSV+ pools of *Culicoides variipennis* complex were sequenced to further identify the species as *C. occidentalis*. Minimum infection rate (MIR) and maximum likelihood estimate (MLE) estimate the total positive individuals per 1000 in the population.

**Table 2:** Temporal and spatial proximity of lesioned rhinoceros and VSNJV-positive vector pools.

Date	Trap	Lesioned Rhinoceros Nearby (n)	VSNJV + Pools (n)	Vector Species
12 June 2023	A	7	2	*C. freeborni; S. vittatum* complex
10 July 2023	A	4	1	*S. hippovorum*
	B	n/a	1	*S. tescorum*
	C	n/a	2	*S. argus; S. tescorum*
	E	6	1	*S. hippovorum*
	F	n/a	1	*C. freeborni*
24 July 2023	A	2	1	*S. hippovorum*

## Data Availability

The data presented in this study will be made available on Ag Data Commons and available by request from the authors.

## Supplementary Materials

The following supporting information can be downloaded at: https://www.mdpi.com/article/10.3390/v16091428/s1, Table S1: Total *Culicoides* biting midge and *Simulium* black fly individuals collected in San Diego County, 2023; Table S2: Pool size, trap ID, Ct values, and CPE Ct values for VSNJV-positive pools; Table S3: Prevalence of NSNJV in pools by species and collection date from *Culicoides* biting midge and *Simulium* black fly individuals collected in San Diego County, 2023.
