# Supplementary material for: Vesicular Stomatitis Virus Detected in Biting Midges and Black Flies during the 2023 Outbreak in Southern California
Source: Viruses. 2024 Sep 7;16(9):1428. doi: 10.3390/v16091428 (PMC11437509; doi:10.3390/v16091428)
Supplement: Supplementary file 1 [file viruses-16-01428-s001.zip › viruses-3181902-supplementary.pdf]

Table S1. Total *Culicoides* biting midge and *Simulium* black fly individuals collected in San Diego County, 2023

Species	5/22	5/29	6/5	6/12	6/19	6/26	7/3	7/10	7/17	7/24	7/31	8/7	8/14	Date unknown	Total
Culicoides bergi	46	20	0	32	18	5	2	0	0	0	2	1	0	2	128
Culicoides crepuscularis	0	0	5	1	0	1	2	0	0	0	0	0	0	0	9
Culicoides freeborni	76	3	44	309	37	232	22	198	0	32	0	105	10	2	1070
*Culicoides variipennis complex**	50	2	26	228	75	170	31	23	0	153	11	348	31	0	1148
Simulium argus	1	1	2	15	3	10	6	7	0	0	0	2	0	0	47
Simulium donovani	19	0	4	9	7	1	5	2	0	0	0	0	0	0	47
Simulium hippovorum	0	0	1	14	1	1	0	8	0	7	0	3	0	0	35
Simulium tescorum	107	25	147	266	189	152	59	34	32	0	0	25	4	10	1050
Simulium vittatum complex	3	0	0	27	4	0	1	1	0	0	0	0	0	0	36

* VSNJV + pools morphologically identified as *Culicoides variipennis complex* were sequenced to further identify the species as *C. occidentalis*.

Table S2. Pool size, Trap ID, Ct values, and CPE Ct values for VSNJV-positive pools

Pool ID	Species	Pool size	Trap ID	Ct	CPE Ct (BHK P1)	CPE Ct (BHK P2)
00274	*Culicoides bergi*	2	29	35.61	Undetermined	Undetermined
00023	*Culicoides freeborni*	7	76	34.77	33.67	Undetermined
00069	*Culicoides freeborni*	1	76	35.59	33.85	Undetermined
00242	*Culicoides freeborni*	5	15	35.59	33.25	Undetermined
00002_7	*Culicoides occidentalis*	5	45	36.04	31.96	Undetermined
00120_1	*Culicoides occidentalis*	6	45	31.11	33.60	Undetermined
00120_2	*Culicoides occidentalis*	5	45	29.09	33.01	Undetermined
00229	*Culicoides occidentalis*	5	54	30.83	33.99	Undetermined
00243	*Culicoides occidentalis*	5	15	36.59	33.67	Undetermined
00064	*Simulium argus*	2	76	35.49	33.51	Undetermined
00052	*Simulium hippovorum*	1	76	28.15	34.64	Undetermined
00071	*Simulium hippovorum*	3	76	35.41	32.66	Undetermined
00112	*Simulium hippovorum*	4	76	36.32	33.53	Undetermined
00047_3	*Simulium tescorum*	4	35	35.34	33.02	Undetermined
00058	*Simulium tescorum*	3	76	36.55	33.65	Undetermined
00063	*Simulium tescorum*	1	76	35.76	32.92	Undetermined
00076	*Simulium tescorum*	1	50	36.39	32.13	35.9
00098	*Simulium tescorum*	6	50	29.19	32.15	Undetermined
00104	*Simulium tescorum*	5	48	33.61	32.04	Undetermined
00117	*Simulium tescorum*	5	55	34.03	32.91	Undetermined
00167	*Simulium tescorum*	5	48	36.15	33.67	Undetermined
00250	*Simulium tescorum*	6	7	34.02	32.51	Undetermined
00004	*Simulium vittatum* complex	1	45	35.33	34.69	Undetermined
00009	*Simulium vittatum* complex	3	21	35.93	30.94	Undetermined
00020	*Simulium vittatum* complex	5	76	35.62	35.59	Undetermined

Table S3. Prevalence of VSNJV in pools by species and collection date from *Culicoides* biting midge and *Simulium* black fly individuals collected in San Diego County, 2023

	Prevalence % (Total + pools, Total pools collected)												
Species	5/22	5/29	6/5	6/12	6/19	6/26	7/3	7/10	7/17	7/24	7/31	8/7	8/14
Culicoides bergi	0	0	0	0	0	0	100 (1,1)	0	0	0	0	0	0
Culicoides freeborni	0	0	0	1.6 (1,64)	0	2.0 (1,49)	0	2.4 (1,42)	0	0	0	0	0
*Culicoides variipennis complex**	8.3 (1,12)	0	0	0	15.8 (3,19)	2.9 (1,35)	0	0	0	0	0	0	0
Simulium argus	0	0	0	0	0	0	0	25 (1,4)	0	0	0	0	0
Simulium hippovorum	0	0	0	0	0	0	0	66.7 (2,3)	0	25 (1,4)	0	0	0
Simulium tescorum	8.9 (2,23)	20.0 (1,5)	2.8 (1,36)	3.4 (2,58)	2.3 (1,43)	0	0	25 (2,8)	0	0	0	0	0
Simulium vittatum complex	33.3 (1,3)	0	0	22.2 (2,9)	0	0	0	0	0	0	0	0	0

* VSNJV + pools morphologically identified as *Culicoides variipennis complex* were sequenced to further identify the species as *C. occidentalis*.
